# Antimicrobial Bioactivity and GC-MS Analysis of Different Extracts of *Corchorus olitorius L* Leaves

**DOI:** 10.1155/2022/3382302

**Published:** 2022-09-20

**Authors:** Rebecca Nakaziba, Sharon Bright Amanya, Crispin Duncan Sesaazi, Frederick Byarugaba, Jasper Ogwal-Okeng, Paul E. Alele

**Affiliations:** ^1^Department of Pharmacology and Therapeutics, Mbarara University of Science and Technology, P.O. Box 1410, Mbarara, Uganda; ^2^Department of Pharmacology and Therapeutics, Lira University, P.O. Box 1035, Lira, Uganda; ^3^Department of Microbiology and Immunology, Lira University, P.O. Box 1035, Lira, Uganda; ^4^Department of Pharmaceutical Sciences, Mbarara University of Science and Technology, P.O. Box 1410, Mbarara, Uganda; ^5^Department of Microbiology, Mbarara University of Science and Technology, P.O. Box 1410, Mbarara, Uganda

## Abstract

**Results:**

Crude extracts of *Corchorus olitorius L* leaves and their TLC-separated components demonstrated bioactivity against *Staphylococcus aureus* (14 mm), *Streptococcus pneumoniae* (16 mm), and *Escherichia coli* (11 mm) but neither against *Candida albicans* nor *Mycobacteria tuberculosis.* However, the overall zones of inhibition were smaller compared to the positive control (≥18 mm). GC-MS analysis of the active components revealed the presence of methyl esters.

**Conclusion:**

*Corchorus olitorius L* is bioactive against both Gram-negative and Gram-positive bacteria but neither against fungi nor mycobacteria. The bioactivity is attributable to the presence of methyl esters. Since methyl esters already have proven bioactivity in some studies, they could be further studied and optimized for possible pharmaceutical use. Further, to provide a more comprehensive antimicrobial spectrum of *Corchorus olitorius L* in Uganda, purified active components could be investigated using a wider range of organisms.

## 1. Introduction

Plants offer an inexhaustible source of bioactive compounds and clinically useful drugs for infectious diseases, cancer as well as cardiovascular disorders [1, 2]. Bioactive compounds play an immense role in drug discovery [3, 4] beginning with bioactivity screening of crude extracts followed by fractionation to isolate the active compounds [5, 6]. Regrettably, there has been limited pharmaceutical development of the plants with known bioactivity with a few pharmaceutical products for infectious diseases [7]. However, the increasing antimicrobial resistance to the currently available antimicrobial agents demands intense investigations into the antimicrobial properties of medicinal plants [8, 9].

*Corchorus olitorius L (C. olitorius*), commonly known as jute mallow, belongs to the genus *Corchorus* and the family of *Tiliceae* [10]. It is a traditional medicinal vegetable worldwide [11]. The vegetable is propagated by seed as well as accepted as a wild plant in crop fields [10]. It has simple, ﬁnely indented leaves and small yellow ﬂowers having ﬁve petals [10]. The vegetable is rich in vitamins A, B_1&2_, C, and *E*; fiber as well as minerals like calcium and iron [12, 13]. The traditional medicinal uses of *C. olitorius* amongst various communities comprise the treatment of heart failure, gastric ulcers, typhoid fever, malaria, gonorrhea, common colds, tumors, and measles [10, 14]. The vegetable also has antiviral [15], antioxidant [16], gastroprotective, and antidiabetic properties [14, 17].

Although *C. olitorius* is used for infectious diseases, there are limited data regarding its antimicrobial spectrum in Uganda. To explore the antimicrobial spectra of *C. olitorius*, we investigated its bioactivity against *Candida albicans* (*C. albicans*), *Staphylococcus aureus* (*S. aureus*), *Streptococcus pneumoniae (S. pneumoniae)*, *Escherichia coli (E. coli)*, and *Mycobacteria tuberculosis* (MTB). *C. olitorius* was active against Gram-positive and Gram-negative bacteria.

## 2. Materials and Methods

### 2.1. Aim, Study Design, and Setting

To investigate the bioactivity of *C. olitorius*, a laboratory-based experimental study was conducted at the Department of Medical Microbiology, Makerere University College of Health Sciences. The plant extraction process was done at the Department of Pharmacology and Therapeutics, Makerere University College of Health Sciences.

### 2.2. Plant Collection and Processing

*C. olitorius* (2 kg) leaves were harvested from Ayer village, Barapwo parish, Lira sub-country, Lira district (Northern Uganda) and taken to the Department of Pharmacology and Therapeutics, Makerere University College of Health Sciences. A voucher specimen was deposited at a herbarium at the Department of Botany, Makerere University Kampala, Uganda (#50906). The leaves were washed clean and spread to dry at room temperature to a constant weight (900 g). After drying, the leaves were ground to powder and extracted using the serial solvent extraction method as described by Das et al. [18]. Diethyl ether, methanol, and distilled water were used as solvents according to their order of polarity. This was done to extract compounds with a wide range of polarity. Briefly, 500 g of ground dried leaves of *C. olitorius* were soaked in 3 L of diethyl ether (BDH AnalaR) and tightly closed in a flat bottomed conical flask while shaking every 6 hours for 48 hours. Thereafter, the resultant solution was filtered using a Whatman filter paper (No. 1) using a funnel and the filtrate was concentrated using a rotary evaporator at 20–30°C. The residue was spread and allowed to dry at room temperature. After which, it was soaked in 3 L of methanol (UltraPure Solutions, Inc. 11485, Commercial Parkway, Castroville, CA 95012) and treated as mentioned above. The last residue was soaked in 3 L of distilled water while shaking every 6 hours for 48 hours and the resultant solution was also filtered using a Whatman filter paper (No. 1) and freeze-dried. After evaporation of the solvents, the extracts were accordingly labeled and stored in a refrigerator at −80°C.

For the bioactivity assay, 0.3 g each of the diethyl ether and methanol crude extracts were dissolved independently in 1 ml DMSO and made up to 10 ml using distilled water while 0.3 g of the aqueous crude extract was dissolved in 10 ml of distilled water to make stock solutions. 100 *μ*l (3000 *μ*g) of each crude extract (stock solution) was used to test for the bioactivity of *C. olitorius* against the organisms used in this study.

### 2.3. The Bioactivity Testing

#### 2.3.1. The Microorganisms

Bacteria included *E. coli* (ATCC 25922); *S. aureus* (ATCC 25923); and *S. pneumoniae* (ATCC 700672) from American Type Culture Collection (Manassas, Virginia, United States). *C. albicans* (wild-type) (Isolated and identified from the medical microbiology laboratory) and MTB (H37Rv) were used.

#### 2.3.2. Culture Media

*E. coli* and *S. aureus* were cultured using Mueller–Hinton agar (MHA) whereas chocolate agar was used to culture *S. pneumoniae*. Sabouraud dextrose agar (SDA) and 7H11 media were used to culture *C. albicans* and MTB, respectively.

#### 2.3.3. Culture Technique

The agar well diffusion technique of culture and sensitivity testing was used [19, 20].

#### 2.3.4. Microbial Culture Procedure

*(1) Bacterial culture*. The culture media containing plates were briefly heated in an oven at 100°C (∼5 minutes) to remove any residual moisture. Colonies of *S. aureus* and *E. coli* were diluted to a turbidity of 0.5 McFarland standard [21] using normal saline and aseptically inoculated on well-labeled culture plates containing MHA. A sterile swab was then used to evenly spread the organisms on the culture plates. Thereafter, wells were dug in the culture plates using a sterile cork (6 mm) and 100 *μ*l (3000 *μ*g) of each crude extract was accordingly pipetted into the wells. *S. pneumoniae* was likewise diluted to 0.5 McFarland standard [21] and aseptically inoculated on culture plates containing chocolate agar media and evenly spread using a sterile swab. Thereafter, wells were dug in the culture plates using a sterile cork (6 mm) and 100 *μ*l (3000 *μ*g) of each extract was accordingly pipetted into the wells. 100 *μ*l of distilled water served as the negative control while chloramphenicol (5 *μ*g) served as a positive control for all tests. Finally, all the culture plates were placed up-right in a CO_2_ incubator at 37°C for 24 hours. At the end of the incubation period, the culture plates were removed and examined for microbial growth inhibition by viewing under natural light. The inhibitory zones were measured in mm using a ruler. All the experiments were performed in duplicates. The crude extracts displayed bioactivity.

*(2) Fungal culture*. To examine the effects of *C. olitorius* extracts on fungal growth, 100 *μ*l (3000 *μ*g) of each extract was independently added to 15 ml of molten SDA; mixed, poured on a well-labeled culture plate, and allowed to solidify. To the negative control plate, neither extract nor *C. albicans* was added while the positive control plate had only agar. Thereafter, the fungi (*C. albicans*) were aseptically and evenly inoculated on all the culture plates (except the negative control) which were thereafter placed in a CO_2_ incubator at 37°C for 18–24 hours. At the end of the incubation period, the culture plates were removed from the incubator and inspected for fungal growth inhibition under natural light. There was no fungal growth inhibition.

*(3) Mycobacterial culture*. To scrutinize the bioactivity of *C. olitorius* against MTB, the crude extracts mentioned above were sterilized using 0.22 *μ*m syringe-driven filters. The 7H11 media plates were brought to room temperature having been stored at 2–8°C and inoculated with 0.5 ml of H37Rv 1.0 McFarland standard strain. Thereafter, wells were dug in the media plates using a cork and 100 *μ*l of each extract was loaded. To the negative control plate, neither H37Rv was inoculated nor extract added whereas for the positive control, the plates were inoculated with H37Rv but no extract was added. All the plates were incubated in a CO_2_ incubator for 21 days and the growth of mycobacteria was inspected. No growth inhibition was exhibited.

### 2.4. TLC Separation of the Crude Extracts of *C. olitorius* Leaves

To identify the components of *C. olitorius* responsible for its bioactivity, the methanolic and diethyl ether crude extracts of *C. olitorius* were subjected to thin layer chromatography (TLC). TLC plates (DC-Fertigfolien ALUGRAMR Xtra SIL G/UV254; MACHERY-NAGEL GmbH & CO. KG-Germany, 20 × 20 cm pre-coated (0.2 mm) with silica gel 60) were cut to a smaller size of 5 × 10 cm using a pair of scissors and used following a method described by Kagan and Flythe [22] with minor modifications. Specifically, instead of using a solvent ratio of 4 : 1 (ethyl acetate (Sigma Aldrich): methanol (BDH)), we used a ratio of 3 : 1 and in place of cutting the separated bands to use them as bio-discs, we scrapped off the bands from the TLC plates and dissolved them in 2 ml of DMSO. The TLC plates (5 × 10 cm) were first cleaned by placing them in a beaker containing the eluent (20 ml). The beaker was covered with an aluminum foil and the eluent was allowed to elute until the solvent front reached the top of the plate. Thereafter, the plates were removed, allowed to dry, and checked for the presence of any bands. The absence of any bands indicated that the plate was clean and ready for use. Then, 5 *μ*l (150 *μ*g) of each extract (stock solution) was independently spotted on the left and right-hand side of the TLC plates upon a line marked using a pencil and allowed to dry. A small volume (50 ml) of the eluent (ethyl acetate: methanol (3 : 1)) was poured into a 500 ml beaker, covered with filter papers, and allowed to saturate. Then, the loaded (spotted) TLC plates were carefully placed into the beaker containing the eluent ensuring that the eluent was below the marked line and that the TLC plates did not touch each other. The beaker was then covered with filter papers and allowed to stand for 20–30 minutes while the elution was taking place. When the solvent front was almost reaching the top of the TLC plates, the plates were removed from the beaker and allowed to dry. The TLC plates were then checked for the presence of any bands visible under natural light. The bands were marked and labeled using a pencil starting with the first band from the spot. Thereafter, these bands were carefully scrapped off the TLC plate and dissolved into 2 ml DMSO. A total of 8 bands (4 TLC plates) of each formed band were dissolved in the DMSO (2 ml).

### 2.5. Bioactivity Testing of the TLC Separated Components

The resultant solutions (following TLC separation) were accordingly labeled and also assayed for bioactivity as described above (see bacterial culture). 100 *μ*l (∼50 *μ*g) of each solution (band) was pipetted into wells dug in the culture plates inoculated with the test bacteria as described above (see bacterial culture). The culture plates were also placed in a CO_2_ incubator for 24 hours and observed for bacterial growth inhibition. The zones of inhibition were measured in mm using a ruler. The components portrayed bioactivity.

### 2.6. GC-MS Analysis of *C. olitorius* Components

To establish the chemical composition of the bands obtained by the TLC that revealed bioactivity, GC-MS analysis was performed. This was done at the Department of Chemistry, Government Analytical laboratories, Wandegeya, Kampala, Uganda, as follows: 100 *μ*l (∼50 *μ*g) of the solutions of the separated *C. olitorius* components were pipetted into separate vials. From each vial, 1 *μ*l (0.5 *μ*g) of the solution was injected into the GC-MS machine (Shimadzu QP2020 NX; Restek column dd5, 0.25 mm thickness) using hydrogen as a carrier gas (1.3 ml/min). The GC oven was held at 80°C and then increased by 4–6°C until 300°C and held for a minimum of 20 minutes. The identification of the compounds in each sample was based upon the mass spectral database in the MS library [23].

### 2.7. Ethical Approval

Permission to conduct the study was secured from the Mbarara University of Science and Technology-Research and Ethics Committee (MUST-REC), The Uganda National Council for Science and Technology (UNCST), and the administrators of the respective departments.

## 3. Results and Discussion

### 3.1. Bioactivity of *C. olitorius* Crude Extracts and Their Components

To assess the bioactivity of *C. olitorius*, the diethyl ether, methanolic, and aqueous crude leaves extracts of *C. olitorius* were tested against *E. coli*, *S. aureus*, *S. pneumoniae*, *C. albicans*, and MTB. *C. olitorius* inhibited bacterial growth but had neither effect on *C. albicans* or MTB (Table 1). *C. olitorius* leaves crude extracts were also separated into individual components using TLC plates and each component was examined for bioactivity. All components displayed bioactivity. However, the overall growth inhibition was less compared to the positive control. Among the crude extracts, the diethyl ether extract exhibited the largest zone of inhibition against *S. aureus* (14.00 mm) and *E. coli* (11.00 mm) whereas the aqueous extract displayed the largest zone of inhibition against *S. pneumoniae* (19.00 mm). The methanolic crude extract showed moderate inhibitory zones against all the bacteria (11.00 mm, 11.50 mm, and 9.50 mm for *S. aureus*, *S. pneumoniae*, and *E. coli*, respectively). The positive control demonstrated the highest inhibition with 20.00 mm, 23.00 mm, and 18.00 mm zones of inhibition for *S. aureus*, *S. pneumoniae*, and *E. coli*, respectively. The negative control did not reveal any inhibition. The results of this study were comparable to the findings of Ilhan and others who conducted a study in Turkey [24] during which different extracts of the *C. olitorius* showed bioactivity against *E. coli, S. aureus,* and *Y. enterocolitica*. In contrast to the findings of Ilhan et al. [24] however, where some fungal species were responsive to the extracts of *C. olitorius* (although most of the fungi were resistant), none of the extracts in the current study demonstrated bioactivity against *C. albicans*. In another study conducted by Sumengen Ozdenefe et al. [25], only the hexane extract demonstrated bioactivity against *S. aureus* and *B. subtilis*. These findings indicate that *C. olitorius* is bioactive against bacteria but has limited activity against fungi. In the current study, the most responsive organism to the inhibitory effects of *C. olitorius* was *S. pneumoniae* with the aqueous extract demonstrating the highest degree of inhibition (19.00 mm) followed by the diethyl ether extract (16.00 mm) while the methanolic extract was the least bioactive (12.00 mm). This finding contradicts Ilhan et al. [24], where the petroleum ether extract of *C. olitorius* was the most bioactive against the organisms they tested. In the study conducted by Sumengen Ozdenefe et al. [25], only the hexane extract of *C. olitorius* demonstrated activity against *B. subtilis*. The other organisms (*S. aureus* and *S. epidermidis*) were resistant to the rest of the extracts (methanol, ethanol, hexane, and chloroform) tested. Nevertheless, the methanolic extract of the current study showed activity against *S. aureus*. Zakaria et al. assayed *C. olitorius* to assess its *in vitro* activity against bacteria and found the methanolic extract active against *C. diphtheriae* and *K. rhizophila* while the chloroform extract demonstrated activity against *S. aureus* [26]. In the current study, all extracts tested showed activity against *S. aureus* including the methanolic extract unlike the findings of Zakaria et al. In related studies by Adegoke and Adebayo et al. [27] and Abir et al. [28] as well as Hayyawi [29], *C. olitorius* demonstrated antibacterial activity comparable to the present study. In the current study, *C. olitorius* demonstrated a broad spectrum nature of antibacterial activity comparable to the findings of Pal et al. [30].

To identify the bioactive components of *C. olitorius* responsible for its bioactivity demonstrated by the crude extracts, the diethyl ether and methanol crude extracts' components were separated using TLC. The diethyl ether extract was separated into 5 (five bands) although the 4th and 5th bands were poorly separated and were thus tested together while the methanol extract was also poorly separated into 2 (two) bands. The separated bands were assayed for bioactivity using the agar well diffusion method as described above (bacterial culture). All components showed antibacterial activity against *E. coli* and *S. pneumoniae*, while only ether bands 1 and 2 showed activity against *S. aureus* (Table 2). Bands ether 1 and 2 demonstrated bioactivity against all the bacteria tested (*S. aureus* (10.00 mm)*, E. coli* (12.00 mm), and *S. pneumoniae* (12.00 mm)). This suggested that the antibacterial activity of the diethyl ether extract against these organisms was due to the components of bands ether 1 and 2. Bands ether 3 to 5, did not show bioactivity against *S. aureus* but against *E. coli* (10.00 mm) and *S. pneumoniae* (12.00 mm). This indicated that these components were responsible for their bioactivity against these organisms. The methanol bands showed bioactivity against only *E. coli* (10.00 mm) and *S. pneumoniae* (12.00 mm). This suggested that bioactivity against *S. aureus* required all components of the methanol extract while bioactivity against *E. coli* and *S. pneumoniae* only required the components present in the bands of the methanolic extract. The bioactive properties of *C. olitorius* can be attributed to the presence of phytochemicals such as alkaloids which have antibacterial activity [31]. The positive control revealed the highest level of growth inhibition while the negative control did not indicate any inhibition in all the bioassays.

### 3.2. Chemical Composition of the Bioactive Component of *C. olitorius* Leaves Extracts

**Figure 1:**
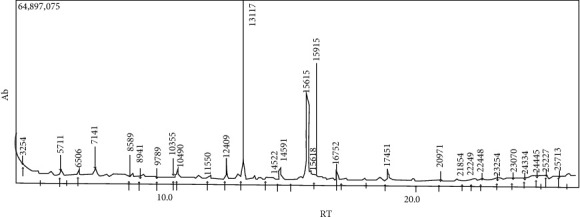
Spectra of the methanol bands. Major composition: hexadecanoic acid, methyl ester (13.117), methyl stearate (15.915), and 9-octadecenoic acid-methyl ester (15.615).

**Figure 2:**
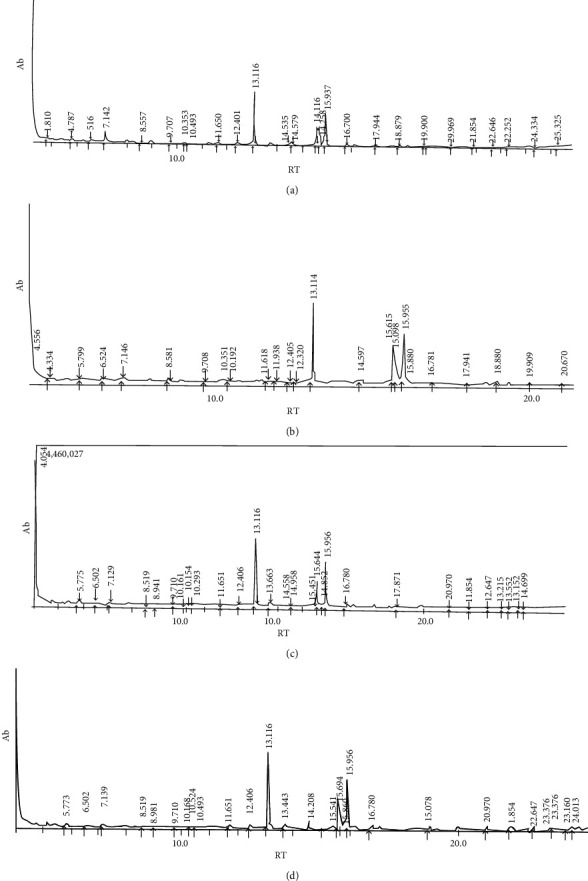
(a) Ether 1: major composition-hexadecanoic acid, methyl ester (13.116), and methyl stearate (15.937). (b) Ether 2: major composition-hexadecanoic acid, methyl ester (13.114), and methyl stearate (15.955). (c) Ether 3: major composition-hexadecanoic acid, methyl ester (13.116), and methyl stearate (15.956). (d) Ether 4 and 5: hexadecanoic acid, methyl ester (13.116), and methyl stearate (15.956).

To identify the chemical composition of the components of *C. olitorius* that demonstrated bioactivity, GC-MS analysis of each bioactive component was performed. The results revealed the presence of methyl esters such as hexadecanoic acid, 9-octadecenoic acid (*z*), and methyl stearate (Figures 1 and 2 and Table 3). The methanol bands revealed the presence of hexadecanoic acid and 9-octadecenoic acid (Figure 1).

The ether bands also revealed the presence of hexadecanoic acid and methyl stearate (Figures 2(a)–2(d)).

#### 3.2.1. Summary of the GC/MS Printouts

In Table 3, the composition of the major peaks revealed by the GC-MS analysis is shown.

The most prominent compound revealed by the GC-MS analysis was methyl stearate. This was in contrast to the findings of Hassan and others who identified cedrane 5-one and *γ*-terpinene as the major bioactive components of *C. olitorius* in a study conducted in Egypt [32]. In addition, a related study by Al-Yousuf et al. [33] showed the presence of hexadecanoic acid and 2,4-di tert butyl phenol in the leaves of *C. olitorius*. This partly concurs with the current study which also indicated the presence of hexadecanoic acid. Further, an additional study by Driss et al. in Tunisia [34] found benzaldehyde to be the major bioactive compound in *C. olitorius* leaves in contrast to the present finding. These differences could be attributed to the locations and the harvest season of the plant. The findings of the current study were partly consistent with those of Hanan and others who found hexadecanoic acid in the leaves of *C. olitorius* [35]. Methyl esters such as hexadecanoic acid, 9-octadecenoic acid, and methyl stearate are reported to have antimicrobial activity [36–38]. In a study conducted by Johannes and Litaay [39], hexadecanoic acid was found to have antibacterial activity. All these findings support the antibacterial activity of *C. olitorius* in the current study.

## 4. Conclusion

*C. olitorius* is bioactive against both Gram-negative and Gram-positive bacteria but neither against fungi nor mycobacteria. It is more active against *S. pneumoniae.* The bioactivity is due to the presence of methyl esters. Since methyl esters have proven bioactivity in some studies, they could be further studied and optimized for possible pharmaceutical use. Further, to provide a more comprehensive antimicrobial spectrum of *C. olitorius* in Uganda, purified active components should be investigated using a wider range of organisms.

## Figures and Tables

**Table 1:** Bioactivity of *C. olitorius* crude leaf extracts; mean zones of inhibition (mm).

*(a) Bioactivity against bacteria*					
Test compound		Concentration in mg/ml	Organism/inhibitory zones (mm)		
			*S. aureus*	*S. pneumoniae*	*E. coli*

*C. olitorius crude extract*	Aqueous	30.00	10.00	19.00	9.00
	Methanol	30.00	11.00	11.50	9.50
	Diethyl ether	30.00	14.00	16.00	11.00
Chloramphenicol		0.05	20.00	22.00	18.00
Distilled water		0.00	0.00	0.00	0.00

*(b) Bioactivity against C. albicans and MTB*					
Test compound		Concentration (mg/ml)	Organism-zone of inhibition (mm)		
			*C. albicans*		MTB

*C. olitorius crude extracts*	Aqueous	30.00	0.00		0.00
	Methanol	30.00	0.00		0.00
	Diethyl ether	30.00	0.00		0.00
Positive control (agar + organism)		0.00	Normal growth		Normal growth
Negative control (neither extract nor organism)		0.00	No growth		No growth

**Table 2:** Bioactivity of *C. olitorius* components; mean zones of inhibition (mm).

Test compound		Concentration in mg/ml	*Organism/inhibitory zones (mm)*		
			*S. aureus*	*S. pneumoniae*	*E. coli*
*C. olitorius* crude extract TLC bands	Ether 1 (*B*)	0.50	10.00	12.00	12.00
	Ether 2 (*Y*)	0.50	10.00	12.00	10.00
	Ether 3 (Br)	0.50	0.00	12.00	10.00
	Ether 4&5 (*Y*)	1.00	0.00	12.00	12.00
	Methanol 1&2 (*G/Y*)	1.00	0.00	12.00	10.00
Chloramphenicol		0.05	20.00	22.00	18.00
DMSO		0.00	0.00	0.00	0.00

*B* = black; *Y* = yellow; Br = brown; G/*Y* = greenish-yellow; PC-positive control; NC-negative control.

**Table 3:** Summary of spectra for methanolic and diethyl ether bands' major peaks.

Sample	Peak *#*	RT	Peak area	Name
*Methanol*				
	15	13.117	150731035	Hexadecanoic acid, methyl ester
	18	15.615	79202331	9-Octadecenoic acid (*z*)-
	21	15.958	96314656	Methyl stearate

*Ether 1*				
	15	13.116	93499849	Hexadecanoic acid
	16	13.661	60793453	Methyl stearate
	20	15.956	40964233	9-Octadecenoic acid (*z*)-, methyl ester

*Ether 2*				
	18	13.114	118125066	Hexadecanoic acid, methyl ester
	20	15.615	60747750	9-Octadecenoic acid (*z*)-, methyl ester
	23	15.955	70655020	Methyl stearate

*Ether 3*				
	13	13.116	117550394	Hexadecanoic acid, methyl ester
	21	15.956	76801423	Methyl stearate
	18	15.614	77813731	9-Octadecenoic acid (*z*)-, methyl ester
	29	23.374	41776224	4-t-Butyl-2-(4-methoxy-phenyl)-6-p-tolyl pyridine

*Ether 4&5*				
	13	13.116	120523802	Hexadecanoic acid, methyl ester
	21	15.956	80539924	Methyl stearate
	18	15.614	56119055	9-Octadecenoic acid (*z*)-, methyl ester

Source: GC-MS printout; Key: RT-retention time.

## Data Availability

The data generated during the study are available from the corresponding author upon reasonable request.

## Abbreviations

*Colitorius*: *Corchorus olitorius* L
DMSO: Dimethyl sulfoxide
TLC: Thin layer chromatography
REC: Research and ethics committee
UNCST: Uganda National Council For Science And technology.
